# Effects of Protein Restriction on Performances and Meat Quality of Cinta Senese Pig Reared in an Organic System

**DOI:** 10.3390/ani9060310

**Published:** 2019-05-31

**Authors:** Chiara Aquilani, Francesco Sirtori, Oreste Franci, Anna Acciaioli, Riccardo Bozzi, Antonio Pezzati, Carolina Pugliese

**Affiliations:** Department of Agri-Food Production and Environmental Sciences, Section of Animal Sciences, University of Firenze, Via delle Cascine 5, 50144 Firenze, Italy; francesco.sirtori@unifi.it (F.S.); oreste.franci@unifi.it (O.F.); anna.acciaioli@unifi.it (A.A.); riccardo.bozzi@unifi.it (R.B.); antonio.pezzati@unifi.it (A.P.); carolina.pugliese@unifi.it (C.P.)

**Keywords:** autochthonous breed, protein content, fat deposition, fatty acids, growing

## Abstract

**Simple Summary:**

Fat contained in meat is an important contributor to sensory traits: it increases meat tenderness and flavor. In commercial pigs, increasing meat’s fat was obtained by feeding, until the fattening period, a diet slightly lower in protein respect to their requirements. Local pig breeds, such as Cinta Senese, are known as obese pigs because their great potential to deposit fat, which is mainly stored in backfat deposits. This study was aimed to assess if protein restriction in growing can further increase meat’s fat without alter overall body fatness of obese pigs. The normal feeding management and the restricted one were compared in two groups of Cinta Senese pigs. Results showed that protein restriction during the growing phase affected only few traits. The restricted animal was more able to use the protein in feeding, but few modifications were found in the chemical composition of meat, including the meat’s fat, that remained unchanged. So, obese genotype might be less responsive to this kind of feeding management.

**Abstract:**

In lean genotypes, protein restriction during growing increases intramuscular fat content without affecting the overall carcass fatness. The present study aims to assess the feasibility of applying this feeding management on an obese pig, the Cinta Senese, since obese genotypes are characterized by great lipogenic potential often leading to excessively high backfat deposits. Twenty pigs of average weight 38 kg, were divided in two groups, the first group was fed a protein restricted diet (9% of crude protein), while the second one a normal diet (13.5% of crude protein). During finishing, both groups were fed the same diet (10% of crude protein). Average daily gain, protein conversion index, backfat thickness, carcass weight, and prime cuts were determined. A loin sample joint was dissected in intermuscular fat, bone, subcutaneous fat, longissimus lumborum, and psoas major. On longissimus lumborum, physical and chemical analysis was carried out. The fatty acid profile of longissimus lumborum and loin subcutaneous fat were determined. Data were analyzed by analysis of variance. Protein conversion index resulted lower in the restricted group, while backfat was slightly greater. Meat quality traits were not affected by feeding management. Slightly modifications in subcutaneous outer layer fatty acids profile were observed. The protein restriction during growing did not seem a suitable mode of feeding management for Cinta Senese pigs.

## 1. Introduction

Feeding management is one of the most known strategies to improve animal performance, meat quality, and eating quality traits in pigs. Diet composition, especially the protein content and the protein:energy ratio during the different rearing phases, deeply affects the carcass composition and the meat quality characteristics, such as the intramuscular fat (IMF) content. Several studies showed that during the growing or finishing phase applying protein or lysine restriction together with an adequate energy supplementation increased the IMF content and improved meat tenderness and juiciness [1,2,3,4]. However, protein restriction during the growing phase also reduces growth rates, slightly increases backfat thickness and carcass fatness, as well as worsens overall carcass traits [5,6]. Suárez-Belloch et al. [7] observed that lysine restriction during the growing phase produced an incomplete compensatory growth in the following period that mainly affected tissues accretion. Indeed, this feeding management seemed to decrease the rate of protein synthesis and increase the energy proportion retained as fat [8,9].

During the last decades, lipid content in meat has been reduced by genetic selection from 2–4% in pork meat of the 1960s to below 1% in the recent leaner genotypes [10]. Though, marbling fat is an important contributor to meat quality, seriously affecting some sensory traits of pork, such as tenderness, juiciness, and flavor. To enhance the pork meat eating quality might be necessary to increase again the IMF content, but, at the same time, this should be performed decreasing the amount of visible fat in order to alleviate consumers health concerns [11]. This is of pivotal importance for native breeds due to their high predisposition to lipogenesis. As for most pig breeds, Cinta Senese, an Italian breed native from Tuscany, shows high lipogenic potential and high predisposition to deposit fat tissue. However, this is mainly stored in the subcutaneous depots, whereas the IMF content of muscle remains unvaried. Moreover, Cinta Senese fattening is usually carried out in Mediterranean silvo-pastoral rearing systems, employing chestnut and acorn, which are very poor in protein and rich in starch and unsaturated fatty acids [12]. This diet enhances the lipogenic attitude of obese genotypes and leads to a higher unsaturation of meat lipid fraction [13,14]. Taking into account these characteristics, the protein restriction followed by a realimentation during the fattening period, could represent an interesting strategy to increase the IMF content of meat and to control or to reduce the subcutaneous fat accumulation. The aim of the study was to investigate the effects of protein restriction on Cinta Senese pigs during the growing phase, followed by a balanced alimentation during the fattening.

## 2. Materials and Methods

### 2.1. Animals and Diets

Twenty Cinta Senese pigs, weighing 38.25 ± 5 kg and 125 days-old, were divided in two dietary groups, balanced per sex (7 barrows and 3 gilts per pen), and allocated in two outdoor fences. During the following 120 days, the first dietary group underwent a protein restriction (RP) by feeding a 9% of crude protein (CP) diet. The second group (NP) was fed a dietary formulation containing 13.5% of CP, usually employed in Cinta Senese farms during the growing phase [15]. At the end of the growing phase (after 120 days, at an average weight of 84 kg), the two groups were fed the same diet containing 10% of CP. Diets are shown in Table 1. Field bean (*Vicia faba* minor) was employed as protein source to address Cinta Senese protected designation of origin (PDO) disciplinary that requires avoiding soybean meal and genetic modified organisms (GMOs). Mineral integration was not used due to the outdoor rearing system applied, in which animals were kept in outdoor paddocks and where they can feed on minerals provided by the soil. The daily feed supplementation was calculated as 90 g/kg of metabolic weight. Animals were weighed every 21 days; in this occasion backfat thickness was assessed by ultrasound and the daily ration was calibrated on the average weight of the group for the subsequent period. Slaughtering took place three times when animals reached the target weight of 150 kg; each time, dietary treatment and sex were adequately represented, as possible. The recording of the in vivo performances stopped when the first group of animals was slaughtered. The trial was carried out in accordance with EU Directive 2010/63/EU and in compliance with the Italian legislation on animal care (DL n. 26, 4 March 2014).

### 2.2. Slaughtering Traits and Meat Quality

Carcasses were weighted six hours after slaughtering, then, on the right side, the prime cuts percentage was assessed and a loin sample joint (LS) (comprehensive of the 2nd and 5th lumbar vertebra and of the surrounding subcutaneous fat with rind) was sampled. After 24 h of chilling (4 °C), the LS was dissected in lean, intermuscular fat, bone, and inner and outer subcutaneous fat; psoas major and longissimus lumborum (LL) muscles were separately weighted. Physical and chemical analysis were carried out on the LL. Instrumental color (L*, a* and b*) was determined by a Minolta Chromameter CR-200 (Tokyo, Japan). Two 20-mm-thick slices of each LL were analyzed at room temperature (22 °C) using a Zwick Roell Z2.5 apparatus (Ulm, Germany) with a loading cell of 1 kN at the crosshead speed of 1 mm/s, to determine texture profile analysis (TPA) considering the following parameters; hardness, cohesiveness, gumminess, springiness, and chewiness. Water-holding capacity (WHC) was evaluated by the free-water method [17], putting the sample (0.3 g) on filter paper, which was placed between two methacrylate plates and pressed at 50 bar for 5 min. Cooking loss was determined by boiling a slice of 20 mm of LL in a water bath until the center temperature reached 75 °C.

Moisture (by lyophilizing to constant weight), total protein, and fat and ash content were determined following AOAC methods [18]. Fatty acids (FAs) of backfat and LL muscle were determined separately. The fatty acid profile was determined using a Varian GC-430 apparatus equipped with a flame ionization detector (FID) (Palo Alto, CA, USA), as reported by Sirtori et al. [19]. The individual methyl esters were identified by their retention time using an analytical standard (F.A.M.E. Mix, C8-C22 Supelco 18,920-1AMP). Response factors based on the internal standard (C19:0) were used for quantification and results were expressed as g/100 g on wet basis.

### 2.3. Statistical Analysis

Data were analyzed by GLM Procedure [20] using the following model.

Y_ijk_ = D_i_ + S_j_ + e_ijk_

where, D = dietary treatment; S = sex; e = random error.

Interaction sex×diet and the effect of the slaughtering day were tested, but, being always not significant, they were not considered in the final model. Level of significance was stated at *p* < 0.05.

## 3. Results and Discussion

In the present study, the dietary restriction applied during the growing phase was a qualitative restriction performed by lowering both the CP content and the Lysine content of the experimental diet (Table 1). The RP and the NP diets supplied a different protein quality, indeed, according the PDO disciplinary followed by most of the Cinta Senese farms, the supplementation of synthetic amino acids is forbidden. This means that, in Cinta Senese rearing systems, protein and lysine restrictions cannot be separated.

### 3.1. In Vivo Performances

Table 2 showed the effect of protein restriction on the in vivo performances of Cinta Senese pigs. The parameters were calculated on the growing phase (until 8 months of age and 84 kg of live weight) and on the following finishing phase, during which animals were fed the same diet (10% CP). At the beginning of the trial, the animals were allocated in the experimental groups according their sex and their live weight to obtain two balanced groups. Indeed, no significant differences were observed between the initial weight of the two groups, as well as between gilts and barrows. Despite the different dietary regimen, no modifications in weight gain, average daily gain (ADG), and backfat thickness were observed at the end of the growing phase. During the following finishing phase any parameters reached the statistical significance The observed values for ADG and backfat thickness are in line with those reposted on previous studies on Cinta Senese [19,21]. However, results on the growing phase are in contrast with those reported by several authors who, in lean genotypes, spotted a clear negative effect of protein restriction on growth performances [6,22,23,24,25]. During the growing phase, only the protein conversion index (PCI) was significantly affected by the diet. Likewise, it approached the statistical significance during the finishing phase with a consistent trend indicating a lower score for RP animals than for NP ones. Considering the growing and finishing phase together, the RP animals showed a lower PCI than NP animals, confirming what observed in the single phases. An improved effectiveness in the amino acids utilization at suboptimal levels was already hypothesized, as well as a prolonged efficiency beyond the end of restriction, that may concur to the compensatory response [26,27].

### 3.2. Carcass Traits

Table 3 shows the results on slaughtering and carcass traits. At slaughter, no differences between RP and NP animals were assessed neither for weight nor for age. After slaughtering, carcass weight and carcass yield were similar for gilts and barrows, but, for the latter parameter, dietary regimen had slightly affected pigs, resulting in a higher carcass yield for NP pigs than for RP ones. Examining the carcass composition in the prime commercial cuts, only backfat resulted affected by diet, being greater in RP animals, whereas carcass yield resulted greater for NP animals. Sex had a greater impact on carcass composition than dietary treatment. Loin was greater in gilts, while backfat was greater in barrows. Consequently, the overall lean cuts were higher in gilts than in barrows, which instead showed a greater percentage of fat cuts. In line with our results, several studies pointed out the greater potential for fattening of barrows compared to gilts [23,24]. The extent of dietary and sex impacts on carcass quality was minor, indeed, focusing on the sample joint, the overall differences observed on carcass disappeared. Outer and inner subcutaneous fat percentages, intermuscular fat, muscles, as well as the overall percentage of lean, fat, and bone, were similar both for dietary groups and animal sex. Considering the dietary amino acid profile, Millet et al. [27] and Fabian et al. [6] suggested that the late finishing diet has the major impact on carcass quality traits, while the amino acid contained in grower and early finishing diets had no effects on carcass, since pigs were able to compensate the restriction when fed a diet with an adequate and balanced amino acidic content. In this study, the late fattening diet supplied the adequate CP level (10%) for Cinta Senese requirements [21]. The slightly greater carcass fatness of RP pigs, suggested by backfat percentage, is in line with several studies reporting how protein shortage during the growing phase causes a complete or partial compensatory growth response during realimentation, also improving the final body fatness [23,28,29]. It is worth noting that most of the studies available in literature were carried out on lean genotypes, or, at least, crossbreed. Considering the high genetic predisposition of obese genotypes to lipid deposition, an adiposity increment, due to the protein shortage in growing, was expected [30]. After a period of protein restriction, several authors suggested that the tissues accretion was modified in favor of fat deposition during realimentation, whereas protein synthesis results reduced [2,8,9]. Despite the increase of backfat, considered as negative, this new pattern of tissue accretion was linked to an enhancement of meat-eating quality thanks to an improvement of IMF content and tenderness [4,25,31]. Indeed, this was the main objective of the present study. Despite the great lipid accretion typical of native breeds, Cinta Senese exerts its genetic potential for lipid deposition mainly increasing the subcutaneous deposits. Likely, considering together the results on carcass traits and on in vivo performances, the dietary protein content both for growing and fattening phases would have been further reduced, given that obese genotypes are lesser responsive to low protein diets than lean genotypes [2,31,32,33] and they had protein requirements rather lower than lean genotypes [15,21,34,35]. Competition at the trough, as well as the great individual variability of Cinta Senese pigs due to the small genetic selection performed on the subjects along decades [36], might be concurrent factors in the great variability observed for most of the examined traits. These could have contributed in failing to reach the significance level for some of the examined parameters.

### 3.3. Meat Quality

Table 4 shows the physical and chemical parameters of fresh loin. Neither grower diet nor sex affected the physical and chemical characteristics of the fresh loin. If the grower protein restriction affected the meat quality, this was overcome in the following period, during which animals were fed the same diet. The results obtained for the physical parameter as well as the chemical composition of LL are in line with earlier studies on Cinta Senese meat quality [21,37]. Madeira et al. [33] reported the dietary protein restriction did not affect the physical meat quality traits in Alentejano pigs. On lean genotypes, Li et al. [25] reported no effect of protein restriction on physical parameter, except for redness, whereas Alonso et al. [10] recorded a slightly lower score for b* in protein- restricted (but not lysine) animals, but no modification for the other physical traits. In both the aforementioned studies, the authors explained the color modifications by the greater IMF content observed in restricted animals, which, in the present study, did not occur. As discussed for carcass traits, obese genotypes genetic features, are supposed to mediate the animal response to protein restriction, making pigs less susceptible to this feeding management compared to lean genotypes [31,33]. Indeed, both Madeira et al. [33] and Liu et al. [38] working, respectively, with Alentejano and Bama mini pig—two obese genotype breeds—observed that the IMF content in LL muscle was affected by genotype but not by protein restriction. Contrariwise, most of the studies carried out on lean genotypes, found that the IMF content of LL muscle increased in the animals fed a protein restricted diet during growing and then realimented in late fattening [10,22,31]. Li et al. [25] reported an increase of the IMF content in pigs fed low CP diets during growing, but especially when applying the protein restriction during the finishing phase. Similarly, Sirtori et al. [21] assessed a greater content of IMF in Cinta Senese pigs fed a 8% CP diet during the whole growing-fattening period. However, in this case, the increase of the IMF was related to an overall worsening of the carcass quality traits that discouraged the formulation of a diet such low in CP. The other tested diets (providing respectively 10, 13, and 16% of CP) did not affect the IMF content, agreeing with the present results. The TPA results (Table 5), were similar among the two dietary groups and between gilts and barrows. The only exception was observed for raw meat cohesiveness, that resulted slightly affect by sex, being lower in gilts than in barrows. Being the IMF content one of the main factors affecting meat tenderness [10], the similar scores obtained by the two dietary groups, especially for the hardiness, are consistent. Considering the positive effect of IMF on meat tenderness and the plausible higher collagen content of Cinta Senese meat due to its generally elevated age at slaughtering, an enhancement of meat marbling would be desirable. However, this was not achieved in the present work.

FAs profiles of lean, inner, and outer subcutaneous fat are shown in Table 6. For brevity, the FAs profile of gilts and barrows are not shown, since the effect of sex was never significant. Lean tissue and backfat inner layer resulted unaffected by dietary treatment, while the major modifications in FAs profile were observed in backfat outer layer. Six FAs showed significant differences, three of them are saturated FAs (SFAs) (C16:0, C18:0, and C20:0) and three are polyunsaturated FAs (PUFAs) (C18:2 n-6, C18:3 n-3, and C22:5 n-3). Except for C20:0 that was greater in RP samples, single FAs were more abundant in NP animals than RP ones and affected also the total SFA and PUFA amounts. Partially agreeing with our results, Suárez-Belloch et al. [23] observed that at decreasing dietary CP levels corresponded a linear decrease of C16:0, C20:0, and C18:3 n-3 and SFA in subcutaneous fat, whereas PUFA showed no differences due to diet. In contrast, Teye et al. [39] found that low protein diets increased the concentration of saturated FAs in subcutaneous fat, while the concentration of PUFA was reduced. The differences observed in subcutaneous outer layer were unexpected, involving both FAs known as products of endogenous synthesis (i.e., C16:0 and C18:0) and FAs related to feeding (i.e., C18:2 n-6 and C18:3 n-3) [40]. Since the animals were fed the same diet for 4 months before slaughtering, the differences observed were not linked to different feeding management.

## 4. Conclusions

In conclusion, the protein restriction during growing, followed by an adequate CP supplementation in fattening, did not affect IMF content, which was the target of the trial. Likely, the protein restriction performed during the growing was not severe enough to obtain a modification of carcass and meat quality traits at slaughtering. The comparable growth performances showed by the two groups, as well as the negligible compensatory response observed in fattening, corroborated this supposition. Overall carcass traits were not affected by protein restriction even if RP pigs resulted proportionally slightly fatter than NP ones, indicating a reduction of lean deposition in favor of fat tissues, even if not IMF one. Among the meat quality traits, only the outer subcutaneous fatty acids profile showed differences among RP and NP animals, with the former samples being lower in SFAs and in PUFAs. To conclude, the applied feeding management might be not suitable for Cinta Senese pigs, which, as most local pig breeds, have lower protein requirements. This could make them lesser responsive to low protein grower diets. Further investigations on large numbers of Cinta Senese pigs are required to determine if IMF deposition could be increased by further reducing the dietary protein content during growing or by extending this feeding management also until fattening.

## Figures and Tables

**Table 1:** Ingredients and chemical composition (% as feed) of the experimental diets.

	Grower Diet (from 4 to 8 Months of Age)		Fattening Diet (from 8 Months to Slaughtering)
	RP	NP	RP and NP
**Ingredients**			
**Barley**	70.00	50.00	50.00
**Field bean**	0.00	25.00	10.00
**Corn**	30.00	25.00	40.00
**Chemical composition ^1^**			
**Crude protein**	9.00	13.49	10.68
**Ether Extract**	2.27	2.12	2.51
**Ash**	2.21	2.47	2.18
**Starch**	53.91	50.55	54.05
**NDF**	28.72	28.10	26.10
**ADF**	6.95	8.68	7.16
**Lys**	0.36	0.68	0.47
**Met**	0.19	0.19	0.19
**Cys**	0.38	0.36	0.34
**Thr**	0.34	0.48	0.38
**Trp**	0.11	0.14	0.11
**Val**	0.48	0.66	0.53
**DE (MJ/kg)**	13.56	13.60	13.73

RP: restricted protein group; NP: normal protein group; DE: digestible energy. ^1^ Chemical composition and amino acids content were determined as sum of the tabulated values of ingredients [16].

**Table 2:** Effects of protein restriction on Cinta Senese pigs: in vivo performances.

	Diet		Sex				
	RP	NP	Gilts	Barrows	RMSE	P Diet	P Sex
***Growing 120 days***							
**Initial weight (kg)**	38.42	37.75	37.19	38.99	5.24	0.771	0.439
**Final Weight (kg)**	82.72	84.58	81.10	86.20	12.64	0.736	0.366
**ADG (g)**	372	393	369	396	72.78	0.505	0.393
**PCI (kg/kg)**	0.48	0.66	0.59	0.55	0.11	0.001	0.427
**Backfat thickness ^1^ (mm)**	1.61	1.49	1.49	1.61	0.44	0.540	0.531
**Inner layer**	0.99	0.84	0.89	0.95	0.31	0.288	0.676
**Outer layer**	0.62	0.65	0.60	0.67	0.19	0.750	0.450
***Finishing 126 days***							
**Initial Weight (kg)**	82.72	84.58	81.10	86.20	12.64	0.736	0.366
**Final weight (kg)**	145.70	142. 20	138.19	149.71	15.27	0.606	0.105
**ADG (g)**	496	443	448	490	59.67	0.058	0.130
**PCI (kg/kg)**	0.62	0.70	0.68	0.63	0.079	0.076	0.090
**Backfat thickness ^1^ (mm)**	1.26	1.49	1.33	1.42	0.45	0.268	0.663
**Inner layer**	0.78	0.95	0.80	0.93	0.36	0.306	0.420
**Outer layer**	0.48	0.54	0.53	0.49	0.20	0.521	0.621
***Growing-finishing 246 days***							
**Initial weight (kg)**	38.42	37.75	37.19	38.99	5.24	0.771	0.439
**Final weight (kg)**	145.70	142. 20	138.19	149.71	15.27	0.606	0.105
**ADG (g)**	436	422	410	448	49.62	0.534	0.103
**PCI (kg/kg)**	0.56	0.67	0.64	0.58	0.07	0.004	0.089
**Backfat thickness ^1^ (mm)**	2.87	3.03	2.83	3.08	0.55	0.525	0.317
**Inner layer**	1.77	1.83	1.69	1.91	0.41	0.755	0.250
**Outer layer**	1.10	1.20	1.13	1.17	0.24	0.360	0.762

RP: restricted protein group; NP: normal protein group; ADG: average daily gain; PCI: protein conversion index (kg of live weight/kg of feed protein); significance level was stated at *p* < 0.05. ^1^ Increase in backfat thickness over the period.

**Table 3:** Effects of protein restriction on Cinta Senese slaughtering traits: postmortem performances on carcass, main cuts and Longissimus l. muscle composition.

	Diet		Sex				
	RP	NP	Gilts	Barrows	RMSE	P Diet	P Sex
**Slaughter weight (kg)**	158.44	151.46	150.95	158.95	9.76	0.130	0.088
**Age at slaughter (days)**	393.80	394.86	399.61	389.04	12.89	0.857	0.087
**Carcass weight (kg)**	130.16	128.83	126.49	132.50	8.73	0.739	0.146
**Carcass yield (%)**	82.20	85.03	83.83	83.40	2.52	0.023	0.708
**Carcass composition (%)**							
Ham	29.24	29.87	30.03	29.08	1.00	0.179	0.051
Shoulder	9.01	8.80	8.85	8.97	0.86	0.595	0.755
Backfat	17.37	15.17	15.15	17.39	1.91	0.020	0.019
Loin	17.02	17.96	18.06	16.92	1.16	0.092	0.045
Belly	11.52	12.02	12.08	11.45	1.88	0.562	0.467
Jowl	3.45	3.31	3.48	3.29	0.50	0.537	0.421
Head	4.20	4.27	4.20	4.27	0.34	0.627	0.676
Trimmed fat	7.41	7.84	7.39	7.86	2.16	0.665	0.636
Foreleg	0.78	0.76	0.77	0.77	0.13	0.802	0.977
Lean cuts	55.28	56.63	56.93	54.97	1.87	0.126	0.033
Fat cuts	39.75	38.34	38.10	39.99	1.94	0.123	0.045
Bone cuts	4.97	5.03	4.97	5.04	0.41	0.746	0.722
**Sample joint composition (%)**							
Total lean	33.36	36.21	35.73	33.85	6.21	0.321	0.511
*-Longissimus lumborum*	20.05	22.46	21.04	21.47	4.62	0.262	0.838
*-Psoas major*	5.95	6.88	6.70	6.14	1.07	0.069	0.263
Total fat	60.71	60.34	58.84	62.22	6.53	0.901	0.267
Subcutaneous fat	58.23	56.11	55.76	58.58	6.08	0.448	0.320
Outer layer	22.80	23.25	22.72	23.33	2.80	0.728	0.634
Inner layer	35.43	32.86	33.04	35.24	4.51	0.222	0.294
Intermuscular fat	2.48	4.24	3.08	3.64	4.21	0.366	0.770
Total bone	5.74	6.81	6.72	5.83	1.45	0.119	0.191

RP: restricted protein group; NP: normal protein group; significance level was stated at *p* < 0.05.

**Table 4:** Physical parameters and chemical composition of fresh loin (% on wet basis).

		Diet		Sex				
		RP	NP	Gilts	Barrows	RMSE	P Diet	P Sex
**WHC (cm^2^)**		11.49	10.84	11.48	10.85	1.96	0.474	0.492
**Cooking loss (%)**		22.41	22.05	22.53	21.93	3.55	0.829	0.723
**Color**	L*	46.19	46.05	45.79	46.44	3.17	0.925	0.668
	a*	14.81	14.48	14.45	14.83	2.06	0.733	0.701
	b*	3.80	3.62	3.57	3.84	1.26	0.758	0.650
**Moisture**		69.87	69.23	69.78	69.32	2.46	0.582	0.685
**Protein**		23.40	23.09	23.28	23.21	1.59	0.684	0.928
**IMF**		5.45	6.66	5.76	6.35	2.33	0.276	0.586
**Ash**		1.11	1.13	1.09	1.16	0.13	0.674	0.254

RP: restricted protein group; NP: normal protein group; WHC: water-holding capacity; significance level was stated at *p* < 0.05.

**Table 5:** Texture profile analysis (TPA) on raw and cooked loin.

	Diet		Sex				
	RP	NP	Gilts	Barrows	RMSE	P Diet	P Sex
**Raw meat**							
**Hardness (N)**	19.56	19.61	21.22	17.95	5.86	0.984	0.233
**Cohesiveness**	0.42	0.41	0.40	0.43	0.02	0.283	0.021
**Springiness**	8.34	8.77	9.07	8.05	1.94	0.629	0.258
**Chewiness (N)**	73.35	74.76	81.35	66.76	35.39	0.931	0.374
**Cooked meat**							
**Hardness (N)**	53.26	51.78	52.48	52.56	10.41	0.756	0.987
**Cohesiveness**	0.58	0.57	0.58	0.57	0.04	0.484	0.843
**Springiness**	9.23	9.32	9.79	8.76	1.15	0.855	0.063
**Chewiness (N)**	293.54	283.67	306.11	271.10	77.27	0.780	0.330

RP: restricted protein group; NP: normal protein group; significance level was stated at *p* < 0.05.

**Table 6:** Fatty acids profile of intramuscular fat of longissimus lumborum (LL) and backfat.

	Intramuscular Fat				Backfat Inner Layer				Backfat Outer Layer			
	RP	NP	RMSE	P Diet	RP	NP	RMSE	P Diet	RP	NP	RMSE	P Diet
**C14:0 ^1^**	0.07	0.07	0.01	0.978	1.29	1.32	0.17	0.715	1.23	1.31	0.10	0.093
**C16:0**	1.29	1.27	0.25	0.898	20.09	20.60	1.41	0.448	18.63	19.56	0.72	0.013
**C16:1 n-7**	0.27	0.27	0.05	0.879	2.02	2.07	0.32	0.762	2.15	2.22	0.16	0.371
**C18:0**	0.49	0.48	0.10	0.863	9.36	9.52	0.88	0.702	7.84	8.40	0.39	0.008
**C18:1 n-9**	2.21	2.19	0.46	0.922	32.53	32.34	2.25	0.857	31.40	32.13	1.78	0.384
**C18:1 n-7**	0.28	0.28	0.05	0.051	2.37	2.36	0.26	0.890	2.48	2.51	0.17	0.690
**C18:2 n-6**	0.21	0.20	0.03	0.374	4.75	5.10	0.47	0.116	5.10	5.65	0.51	0.034
**C18:3 n-3**	0.01	0.01	0.00	0.728	0.30	0.33	0.04	0.179	0.32	0.40	0.05	0.004
**C20:0**	0.01	0.01	0.00	0.871	0.13	0.13	0.02	0.875	0.40	0.12	0.01	0.049
**C20:1 n-9**	0.03	0.03	0.01	0.664	0.69	0.69	0.10	0.623	0.69	0.69	0.06	0.916
**C20:2 n-6**	0.00	0.00	0.00	0.974	0.22	0.23	0.03	0.707	0.25	0.27	0.03	0.287
**C20:3 n-6**	0.01	0.00	0.00	0.210	0.03	0.03	0.00	0.336	0.03	0.03	0.00	0.426
**C20:4 n-6**	0.04	0.04	0.01	0.253	0.06	0.06	0.01	0.487	0.06	0.07	0.01	0.185
**C20:3 n-3**	0.00	0.00	0.00	0.772	0.08	0.07	0.01	0.690	0.09	0.10	0.01	0.311
**C20:5 n-3**	0.00	0.00	0.00	0.474	0.00	0.00	0.00	0.615	0.00	0.00	0.00	0.250
**C22:0**	0.00	0.00	0.00	0.514	0.00	0.00	0.00	0.642	0.00	0.00	0.00	0.364
**C22:1 n-9**	0.00	0.00	0.00	0.464	0.01	0.01	0.00	0.192	0.01	0.01	0.00	0.424
**C22:4 n-6**	0.00	0.00	0.09	0.405	0.02	0.02	0.00	0.963	0.03	0.03	0.00	0.687
**C22:5 n-3**	0.00	0.00	0.00	0.697	0.02	0.02	0.00	0.935	0.02	0.02	0.00	0.026
**Total SFA**	1.87	1.85	0.36	0.883	31.42	32.05	2.14	0.530	28.33	29.93	1.10	0.006
**Total MUFA**	2.84	2.80	0.56	0.889	38.29	38.02	2.62	0.826	37.36	38.22	2.09	0.395
**PUFA n-3**	0.02	0.02	0.00	0.781	0.39	0.42	0.06	0.308	0.44	0.52	0.06	0.010
**PUFA n-6**	0.27	0.25	0.04	0.344	5.07	5.44	0.49	0.132	5.47	6.04	0.54	0.038
**n-6/n-3**	14.38	12.75	1.95	0.091	13.10	12.98	1.16	0.827	12.60	11.70	1.26	0.142
**Total PUFA**	0.29	0.27	0.04	0.400	5.50	5.89	0.54	0.142	5.95	6.59	0.58	0.028

RP: restricted protein group; NP: normal protein group; significance level was stated at *p* < 0.05. Single FAs were expressed as g/100 g of tissue (lean or backfat layers). ^2^ Trace amounts of the following FAs were also identified; C12:0, C13:0, C14:1n5, C15:0, C16:1n9, anteisoC17:0, C17:0, C17:1, C20:1n7, and C18:3n4. For brevity, they were included in the total sums, but not shown in the table.
